# Research progress of HIF-1a on immunotherapy outcomes in immune vascular microenvironment

**DOI:** 10.3389/fimmu.2025.1549276

**Published:** 2025-02-06

**Authors:** Shaoyan Shi, Xuehai Ou, Chao Liu, Hao Wen, Jiang Ke

**Affiliations:** Department of Hand Surgery, Honghui Hospital, Xi’an Jiaotong University, Xi'an, China

**Keywords:** HIF-1 α, VEGF, immune vascular microenvironment, immunotherapy resistance, personalized cancer therapy, machine learning HIF-1a, immune vascular microenvironment and immunotherapy

## Abstract

The hypoxia-inducible factor-1α (HIF-1α) plays a key role in facilitating the adaptation of cells to hypoxia, profoundly influencing the immune vascular microenvironment (IVM) and immunotherapy outcomes. HIF-1α-mediated tumor hypoxia drives angiogenesis, immune suppression, and extracellular matrix remodeling, creating an environment that promotes tumor progression and resistance to immunotherapies. HIF-1α regulates critical pathways, including the expression of vascular endothelial growth factor and immune checkpoint upregulation, leading to tumor-infiltrating lymphocyte dysfunction and recruitment of immunosuppressive cells like regulatory T cells and myeloid-derived suppressor cells. These alterations reduce the efficacy of checkpoint inhibitors and other immunotherapies. Recent studies highlight therapeutic strategies that target HIF-1α, such as the use of pharmacological inhibitors, gene editing techniques, and hypoxia-modulating treatments, which show promise in enhancing responses to immunotherapy. This review explores the molecular mechanisms of action of HIF-1α in IVM, its impact on immunotherapy resistance, as well as potential interventions, emphasizing the need for innovative approaches to circumvent hypoxia-driven immunosuppression in cancer therapy.

## 1 Introduction

Hypoxia-inducible factor-1α (HIF-1α) is a key regulator of cellular responses to oxygen deprivation, an essential aspect of physiological and pathological processes (1–3). Under normal oxygen levels (normoxia), HIF-1α is hydroxylated by prolyl hydroxylase domain enzymes (PHDs), causing its ubiquitination and subsequent degradation via the von Hippel-Lindau (VHL)-proteasome pathway. In contrast, HIF-1α is stabilized under hypoxic conditions, enabling its dimerization with HIF-1β and translocation to the nucleus, where it activates a series of genes crucial for adaptive responses (4, 5). The stabilization of HIF-1α triggers downstream signaling pathways regulating processes such as angiogenesis, glycolysis, erythropoiesis, and cellular survival. This transcription factor has significant roles in tumor biology, as hypoxia is a characteristic of the tumor microenvironment (TME). Within tumors, hypoxic regions arise due to rapid cell proliferation and insufficient vascular supply, compelling cancer cells to adapt through the activation of HIF-1α. This adaptation promotes angiogenesis by upregulating vascular endothelial growth factor (VEGF), a critical driver of aberrant vasculature in tumors (6, 7). Additionally, HIF-1α facilitates metabolic reprogramming, enabling cancer cells to utilize anaerobic glycolysis even in oxygen-limited conditions (8, 9). The influence of HIF-1α extends beyond tumor cells to other components of the tumor microenvironment, including immune cells and stromal elements. For instance, HIF-1α regulates the recruitment and polarization of macrophages, driving them towards an immunosuppressive M2 phenotype that promotes tumor progression. Similarly, it interferes with cytotoxic T lymphocytes (CTLs) by promoting an immunosuppressive environment, thereby facilitating immune evasion (10, 11). These multifaceted roles position HIF-1α as a key player in cancer progression and resistance to therapy, particularly in hypoxia-driven conditions.

Within the immune vascular microenvironment (IVM) of the TME, a dynamic interplay occurs between immune cells, vasculature, and the extracellular matrix (ECM) (12, 13). This complex network governs immune surveillance, nutrient delivery, and cellular interactions, critically impacting tumor progression and therapeutic outcomes. The IVM consists of several immune cells, including T lymphocytes, macrophages, natural killer (NK) cells, dendritic cells, and myeloid-derived suppressor cells (MDSCs), alongside endothelial cells and pericytes that form the vasculature. The ECM serves as a structural scaffold concurrently regulating cell signaling and immune cell infiltration. These components in tumors are often dysregulated, developing an immunosuppressive and pro-tumorigenic environment.

Hypoxia profoundly influences the IVM, primarily through the activation of HIF-1α, which drives angiogenesis by upregulating VEGF, resulting in the formation of irregular, leaky blood vessels that disrupt effective immune cell infiltration (14, 15). The aberrant vasculature also fosters hypoxia, establishing a feedback loop that sustains HIF-1α activity. Hypoxia shapes the immune landscape by altering immune cell recruitment and function. For example, it polarizes macrophages towards the M2 phenotype, diminishes the cytotoxic activity of CD8+ T cells, and promotes the accumulation of T regulatory cells (Tregs) and MDSCs, all of which facilitate immune evasion (16, 17). The ECM within the IVM also undergoes hypoxia-induced remodeling, under the action of HIF-1α-regulated matrix metalloproteinases (MMPs). This remodeling creates physical barriers that impede immune cell infiltration while releasing bioactive molecules that promote angiogenesis and immune suppression. These factors collectively create a microenvironment that undermines immune surveillance and drives tumor progression, presenting significant challenges for immunotherapy.

Immunotherapy, harnessing the body’s immune system to target and eliminate tumors, has emerged as a transformative approach in cancer treatment. Key strategies include the use of immune checkpoint inhibitors (ICIs), chimeric antigen receptor (CAR)-T cell therapy, and cancer vaccines, each targeting specific aspects of immune regulation (18, 19). ICIs, including anti-PD-1/PD-L1 and anti-CTLA-4 antibodies, function by reactivating exhausted T cells, enabling them to recognize and attack cancer cells. While ICIs have shown remarkable success in certain cancers, their efficacy is often limited in hypoxic and immunosuppressive TMEs. Unser hypoxia, HIF-1α upregulates PD-L1 expression, contributing to T cell exhaustion and resistance to ICIs. CAR-T cell therapy involves engineering T cells to express CARs that target tumor-specific antigens (20, 21). Although effective in hematological malignancies, the activity of CAR-T cells is challenging in solid tumors due to the hypoxic and immunosuppressive IVM. HIF-1α alters the chemokine landscape, impairing trafficking and survival of CAR-T cells within the TME. Cancer vaccines aim to stimulate immune responses against tumor-specific antigens. However, the hypoxic IVM limits the efficacy of vaccine by impairing antigen presentation and fostering an immunosuppressive environment.

Hypoxia and the resulting HIF-1α-driven changes in the IVM exert significant barriers to the success of these therapies. In addition to the abnormal vasculature limiting immune cell infiltration, and the accumulation of immunosuppressive cells like Tregs and MDSCs further dampening therapeutic responses, hypoxia-induced metabolic changes, such as increased lactate production, create an acidic microenvironment further hindering immune cell function. With these challenges, addressing the role of HIF-1α in the IVM is critical to enhancing the efficacy of immunotherapies. Strategies, such as targeting HIF-1α or normalizing the vasculature, to overcome hypoxia-driven immunosuppression are under active investigation and hold promise for improving therapeutic outcomes.

The complex interaction between hypoxia, HIF-1α, and the IVM represents a critical axis influencing cancer progression and therapeutic resistance. While immunotherapy has revolutionized cancer treatment, its efficacy is often undermined by the hypoxic and immunosuppressive conditions prevalent in the IVM. HIF-1α emerges as a key mediator in this context, orchestrating changes in vascular dynamics, immune cell function, and ECM remodeling that collectively promote immune evasion. This review aims to provide a comprehensive overview of the role of HIF-1α in shaping the IVM and its implications for immunotherapy outcomes. This work elucidates the molecular mechanisms through which HIF-1α drives hypoxia-induced changes, and seeks to highlight potential therapeutic interventions targeting this pathway. The review explores current strategies to inhibit HIF-1α or modulate the hypoxic microenvironment, with an emphasis on their integration into existing immunotherapy regimens. Through this analysis, the review aims to serve as a resource for researchers and clinicians, offering insights into the complex biology of the IVM and its implications for therapeutic innovation.

## 2 HIF-1α: molecular mechanisms and regulation

### 2.1 Structure and activation

HIF-1α is a heterodimeric transcription factor that plays a key role in cellular adaptation to low oxygen (hypoxia) conditions. Structurally, it comprises two subunits: HIF-1α and HIF-1β (also known as aryl hydrocarbon receptor nuclear translocator [ARNT]). While HIF-1β is constitutively and stably expressed under normoxic and hypoxic conditions, the activity of HIF-1α is tightly regulated by oxygen availability. The HIF-1α protein consists of several functional domains: the basic helix-loop-helix (bHLH) domain, which is critical for DNA binding and dimerization with HIF-1β (22, 23); the per-ARNT-Sim (PAS) domain, which facilitates heterodimerization and stabilization of the HIF-1 complex (24, 25); the oxygen-dependent degradation domain (ODD), which serves as the regulatory domain for oxygen-sensitive degradation (26, 27); and transactivation domains (TADs), TAD-N and TAD-C, which are responsible for transcriptional activation of downstream target genes (28).

Under normoxic conditions, proline residues in the HIF-1α ODD domain is hydroxylated by PHDs (29, 30). This hydroxylation promotes the binding of the ubiquitin ligase VHL E3 complex to HIF-1α, leading to its ubiquitination and subsequent proteasomal degradation. Further, under normoxia, the factor inhibiting HIF-1α (FIH) hydroxylates an asparagine residue within the TAD-C domain, preventing the recruitment of coactivators and transcriptional activation. Under hypoxic conditions, PHDs and FIH activities are inhibited due to a lack of molecular oxygen, an essential cofactor for these enzymes. Consequently, HIF-1α stabilizes, translocates to the nucleus, and dimerizes with HIF-1β. The HIF-1 complex then binds to hypoxia-responsive elements (HREs) at target gene promoters, initiating their transcription (31, 32). This oxygen-sensitive regulation allows HIF-1α to function as a molecular sensor and effector, orchestrating cellular adaptation to hypoxia.

### 2.2 Downstream targets of hypoxia-inducible factor-1α

HIF-1α regulates the expression of a wide array of genes participating in critical processes such as angiogenesis, metabolism, and immune modulation. Under hypoxic conditions within tumors, HIF-1α induces the expression of VEGF, a potent angiogenic factor that promotes endothelial cell proliferation and migration (8, 9). VEGF plays a key role in angiogenesis, the formation of new blood vessels,. However, the vasculature formed is often disorganized and leaky, leading to a hostile IVM (33, 34). HIF-1α drives metabolic reprogramming by upregulating enzymes involved in glycolysis, such as hexokinase 2, phosphofructokinase, and lactate dehydrogenase A (35, 36). This shift towards anaerobic glycolysis, known as the Warburg effect, promotes cancer cells to survive and thrive in hypoxic environments while producing lactate, which further acidifies the TME and suppresses immune cell function. HIF-1α directly influences immune evasion by regulating immune checkpoint molecules, including programmed death-ligand 1 (PD-L1). Hypoxia-induced expression of PD-L1 on tumor cells and immune cells impairs T cell activity, leading to immunosuppression (37, 38). HIF-1α also supports the expression of other inhibitory receptors, including CTLA-4 and LAG-3. HIF-1α induces MMPs, such as MMP-2 and MMP-9, which degrade the ECM (39, 40). This process facilitates tumor invasion and metastasis while altering the ECM composition to create physical barriers to immune cell infiltration.

### 2.3 Crosstalk with other signaling pathways

**Figure 1:**
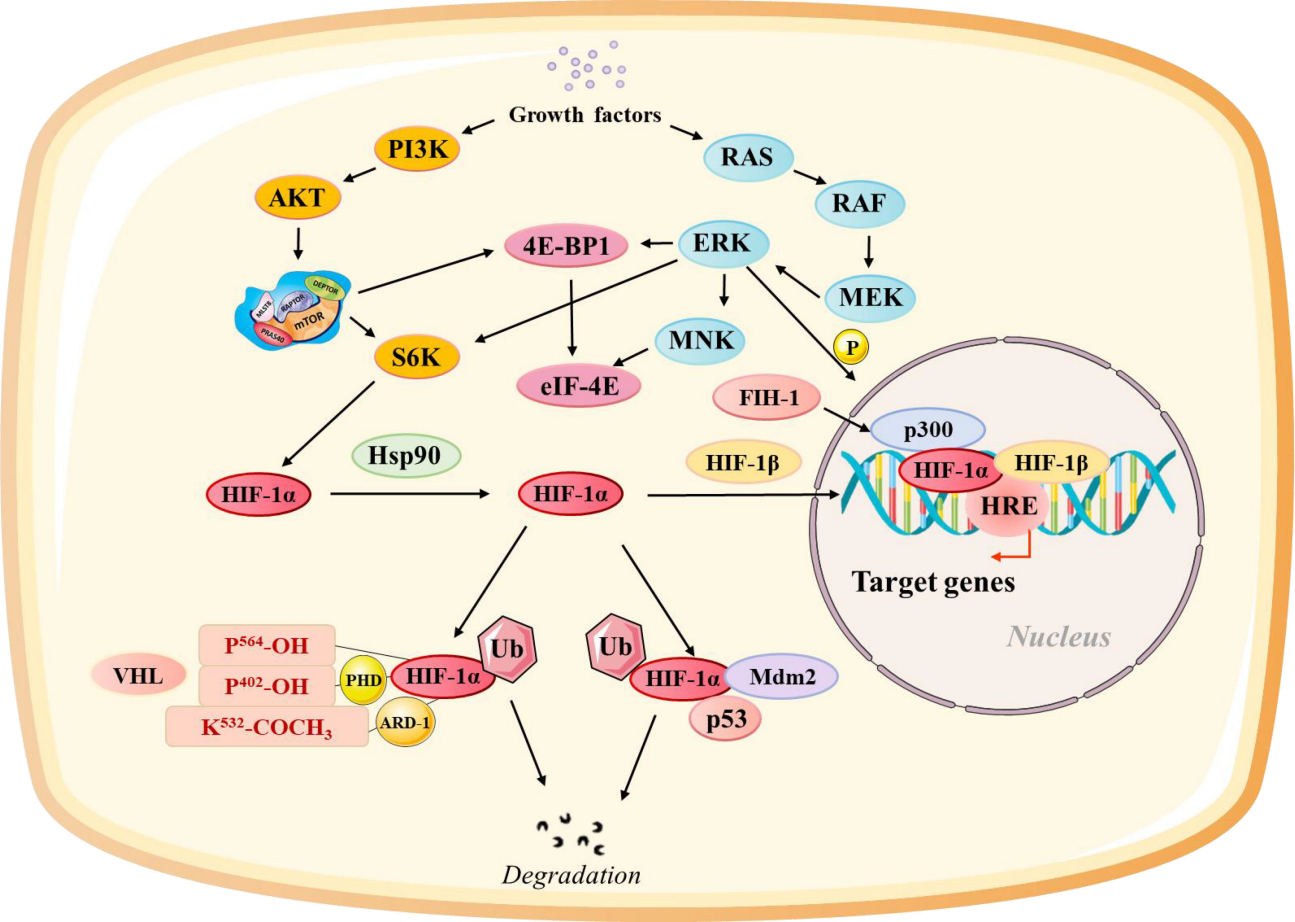
Regulation of the Hypoxia-Inducible Factor Alpha (HIF-1α) Pathway at Different Levels. HIF-1α activity is modulated through multiple regulatory pathways: Growth factor-related pathways influence the synthesis of HIF-1α through the rat sarcoma/rapidly accelerated fibrosarcoma/MAPK/ERK kinase (Ras/Raf/MEK). pVHL-related pathways control the stability of HIF-1α through oxygen-dependent degradation. Factor inhibiting HIF-1α (FIH-1) regulates HIF-1α transactivation by hydroxylating its transactivation domain. The Mdm2-p53 pathway mediates ubiquitination and proteasomal degradation of HIF-1α. Heat shock protein 90 (HSP90) stabilizes HIF-1α and facilitates its transactivation.

HIF-1α interacts with multiple signaling pathways, with its effects amplified and cellular responses integrated into environmental and metabolic changes (**Figure 1**): The mammalian target of rapamycin (mTOR) pathway is a central regulator of cell growth and metabolism. Hypoxia-induced mTOR activation enhances the rate of HIF-1α transcription and translation, creating a feed-forward loop that strengthens the hypoxic response (41, 42). Conversely, the inhibition of mTOR reduces HIF-1α levels and dampens the transcription of its downstream targets, highlighting the interplay between oxygen sensing and nutrient signaling (8). Signal transducer and activator of transcription 3 (STAT3) is a key mediator of inflammation and tumor progression. HIF-1α and STAT3 interact to upregulate VEGF and promote angiogenesis (43, 44). Furthermore, HIF-1α-induced STAT3 activity enhances the immunosuppressive functions of tumor-associated macrophages (TAMs) and Tregs, contributing to immune evasion.

A transcription factor, nuclear factor kappa-light-chain-enhancer of activated B cells (NF-κB), responds to inflammatory and stress signals (45, 46). HIF-1α stabilizes NF-κB under hypoxia, causing the expression of pro-inflammatory cytokines, such as interleukin-6 (IL-6) and tumor necrosis factor-alpha. This crosstalk promotes a chronic inflammatory state that supports tumor progression and alters immune cell recruitment and activation. HIF-1α further interacts with the Notch and Wnt pathways to regulate cancer cell stemness and differentiation. These interactions also influence immune cell recruitment and function, further shaping the IVM. The crosstalk between HIF-1α and these signaling pathways highlights its central role in integrating diverse environmental signals, enabling tumors to adapt to hypoxia while evading immune surveillance.

### 2.4 Hypoxia-induced changes in immune vascular microenvironment mediated by hypoxia inducible factor-1α

The IVM is profoundly affected by the hypoxia-driven activity of HIF-1α, impacting angiogenesis, immune cell infiltration, and ECM remodeling; HIF-1α-induced VEGF expression drives abnormal angiogenesis, characterizing disorganized, leaky blood vessels (47, 48). These vessels fail to adequately oxygenate tissues, perpetuating hypoxia and the activity of HIF-1α in a vicious cycle. The resulting vascular abnormalities hinder effective transport of immune cells to the tumor, creating “immune exclusion zones” that prevent cytotoxic T cells from reaching their targets. Hypoxia alters immune cells’ behavior and their recruitment within the IVM. HIF-1α promotes the polarization of macrophages towards the M2 phenotype, which supports tumor growth and suppresses immune responses. It also enhances the recruitment of immunosuppressive cells, such as MDSCs and Tregs, concurrently impairing the function of effector cells like CD8+ T cells and NK cells (10, 49). These changes cumulatively contribute to an immunosuppressive microenvironment.

HIF-1α regulates MMPs and other enzymes that remodel the ECM, creating structural barriers to immune cell infiltration. ECM remodeling also releases sequestered growth factors, further supporting angiogenesis and tumor progression. Hypoxia-induced activity of HIF-1α leads to enhanced expression of immune checkpoint molecules like PD-L1, promoting T cell exhaustion (37, 50). HIF-1α also drives metabolic changes that favor lactate accumulation and acidosis, suppressing immune cell activation and function. These HIF-1α-mediated hypoxia-induced changes present significant challenges for immunotherapy. Targeting HIF-1α or its downstream pathways offers a potential strategy for IVM reprogramming, normalized vasculature, and enhanced immune cell function, thereby improving the efficacy of immunotherapeutic interventions.

HIF-1α is a central regulator of hypoxia-induced changes in the TME, with far-reaching effects on angiogenesis, immune modulation, and ECM remodeling. Its activation and downstream signaling pathways orchestrate a coordinated response to oxygen deprivation, enabling tumors to thrive in hypoxic conditions while evading immune surveillance.

## 3 Immune vascular microenvironment in cancer

The IVM is a dynamic and intricate component of the TME, characterizing the interplay of immune cells, blood vessels, and the ECM. These elements are profoundly affected by hypoxia, a hallmark of solid tumors, and contribute to tumor progression, immune evasion, and therapeutic resistance. This section explores the defining features of the IVM, immune cell behavior in hypoxic conditions, and the potential benefits of vascular normalization in overcoming the challenges posed by the tumor IVM.

### 3.1 Defining features of immune vasculature microenvironment in tumors

The IVM in tumors characterizes abnormal vasculature, immune cell exclusion zones, and hypoxia-induced immunosuppression. These features create a conducive microenvironment for tumor growth and immune evasion.

Abnormal structure and function are a few hallmarks of tumor vasculature. The rapid proliferation of tumor cells triggers an overexpression of pro-angiogenic factors, including VEGF, largely regulated by HIF-1α (51, 52). This results in abnormal and leaky blood vessels that are unable to provide adequate oxygen and nutrients to the tumor (53, 54). The resulting hypoxic conditions perpetuate a cycle of HIF-1α activation, further exacerbating vascular abnormalities. These disorganized vessels also impair efficient trafficking of immune cells into the tumor, leading to the formation of immune cell exclusion zones. CTLs and NK cells, which are crucial for tumor cell killing, unable to penetrate the dense stroma, often remain confined to the tumor periphery.

Hypoxia-induced immunosuppression further compounds these challenges. Through the activation of HIF-1α, hypoxia alters immune responses by promoting the expression of immune checkpoint molecules such as PD-L1 on tumor and immune cells, leading to T cell exhaustion (55, 56). Additionally, hypoxia enhances the recruitment and function of immunosuppressive cells, including Tregs, MDSCs, and M2-polarized macrophages. These features collectively create an IVM that fosters tumor progression while resisting immune surveillance and therapeutic interventions.

### 3.2 Immune cell behavior in the hypoxic immune vasculature microenvironment

Hypoxia significantly influences immune cell behavior and function within the IVM, altering their roles in anti-tumor immunity and contributing to immune evasion. Macrophages exhibit a high degree of plasticity and are among the most abundant immune cells in the IVM. They can polarize into two functional phenotypes: the pro-inflammatory, anti-tumor M1 phenotype or the immunosuppressive, pro-tumor M2 phenotype. Hypoxic conditions driven by HIF-1α shift macrophages toward the M2 phenotype by upregulating factors such as VEGF, IL-10, and arginase-1 (57, 58). These M2 macrophages support tumor progression by promoting angiogenesis, suppressing CTL activity, and remodeling the ECM to facilitate tumor invasion and metastasis (59). In contrast, the pro-inflammatory functions of M1 macrophages, such as their ability to produce cytokines and reactive oxygen species (ROS) that kill tumor cells, are largely suppressed in the hypoxic IVM. Reprogramming macrophages from the M2 to M1 phenotype is an area of active research aimed at enhancing anti-tumor immunity.

The function of CTLs and NK cells, the primary effectors of anti-tumor immunity, is also severely compromised in the hypoxic IVM. Hypoxia-induced HIF-1α upregulates PD-L1 expression on tumor cells and antigen-presenting cells, leading to T cell exhaustion—a state characterized by reduced proliferation, cytokine production, and cytotoxicity. Hypoxia also alters the chemokine landscape of the TME, downregulating the expression of factors like CXCL9 and CXCL10 that are essential for T cell recruitment. Likewise, hypoxia also negatively affects NK cells. HIF-1α reduces the expression of activating receptors on NK cells, impairing their ability to recognize and kill tumor cells. The accumulation of lactate in the hypoxic tumor environment further inhibits NK cell function, reducing their production of interferon-gamma and cytotoxic granules (60, 61). These immune cell dysfunctions collectively create a profoundly immunosuppressive IVM that supports tumor growth and undermines the efficacy of immunotherapies. Understanding these mechanisms is critical for developing strategies to restore immune cell activity in the TME.

### 3.3 Vascular normalization hypothesis

According to this hypothesis, restructuring the abnormal tumor vasculature to resemble normal vasculature more closely can alleviate hypoxia, reduce immunosuppression, and enhance the efficacy of immunotherapies. This approach has gained traction to address the challenges posed by the disorganized and dysfunctional vasculature in tumors. Vascular normalization aims to create functional, evenly distributed blood vessels that improve oxygenation and facilitate the infiltration of immune cells into the tumor. By reducing hypoxia, vascular normalization decreases HIF-1α activity and its downstream effects on immunosuppression. This results in a more favorable microenvironment for immune cell activity and less conducive to tumor progression.

The normalization of tumor vasculature for immunotherapy has several advantages. First, it enhances the infiltration of CTLs, NK cells, and other effector immune cells into the tumor core, thereby increasing the likelihood of effective anti-tumor responses. This is particularly critical for ICIs, which rely on CTLs within the tumor to unleash their cytotoxic activity (10, 62). Second, improved oxygenation reduces hypoxia-driven immunosuppressive mechanisms, such as lactate accumulation and immune checkpoint expression. Finally, vascular normalization, by reducing interstitial fluid pressure and improving drug distribution within the tumor, optimizes the delivery of immunotherapeutic agents, such as monoclonal antibodies and adoptive cell therapies.

Several strategies for achieving vascular normalization have been explored. Anti-angiogenic therapies targeting VEGF, such as bevacizumab, have shown promise in providing temporary normalization of tumor vasculature and enhancing the delivery of immune cells and drugs. However, excessive VEGF inhibition can lead to vessel regression and exacerbate hypoxia, underscoring the need for balanced approaches. Other strategies, such as targeting angiopoietin-2 (Ang-2) or using blood vessel stabilizing agents, have shown potential in promoting vascular normalization (63, 64). Combining vascular normalization agents with immunotherapies has demonstrated synergism in preclinical and clinical studies, paving the way for innovative combination therapies.

The IVM in cancer is shaped by abnormal vasculature, immune cell exclusion zones, and hypoxia-induced immunosuppression, all of which hinder effective anti-tumor immunity and therapeutic outcomes. Advances in vascular normalization, particularly in combination with immunotherapies, hold significant potential for transforming cancer treatment by overcoming the unique challenges of the tumor IVM.

## 4 Impact of hypoxia inducible factor-1α on immunotherapy

**Figure 2:**
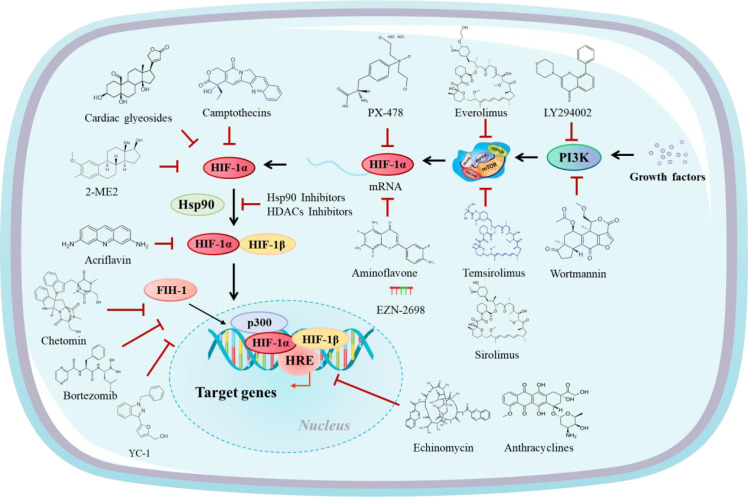
HIF-1α inhibitors modulate different levels of the HIF-1α activation pathway.

HIF-1α profoundly influences the immune response and therapeutic outcomes in cancer by shaping the TME. This section explores the mechanisms through which HIF-1α impacts immunotherapy, focusing on its role in immune checkpoint regulation, modulation of tumor-infiltrating immune cells, resistance to immunotherapy, and potential therapeutic strategies targeting HIF-1α (**Figure 2**).

### 4.1 Influence on immune checkpoints

HIF-1α plays a key role in upregulating immune checkpoint molecules, such as PD-L1, which are critical mediators of immune evasion. In the hypoxic TME, HIF-1α directly binds to the HREs within the promoter regions of PD-L1, enhancing its transcription. Elevated PD-L1 expression on tumor cells and antigen-presenting cells interacts with programmed death-1 (PD-1) on T cells, leading to T cell exhaustion (50, 65). Exhausted T cells possess reduced proliferative capacity, diminished cytokine production, and impaired cytotoxic activity, allowing tumor cells to escape immune surveillance. Beyond PD-L1, HIF-1α also regulates other inhibitory molecules, such as CTLA-4 and lymphocyte activation gene-3 (LAG-3), which further suppress T cell activity. Additionally, HIF-1α upregulates galectin-9 and its ligand, TIM-3, which contribute to the exhaustion of CD8+ T cells and the expansion of Tregs.

### 4.2 Modulation of tumor-infiltrating immune cells

HIF-1α profoundly alters the composition and behavior of tumor-infiltrating immune cells, altering the immune response towards immunosuppression and tumor progression. Hypoxia and HIF-1α impair the function of CD8+ T cells, the primary effectors of anti-tumor immunity (5, 66). HIF-1α-mediated upregulation of PD-L1 on tumor cells and antigen-presenting cells inhibits T cell activation, leading to exhaustion. The acidic pH reduces the production of key cytokines like IFN-γ, which is essential for effective cytotoxic responses. These metabolic barriers suppress CD8+ T cell activity and also promote their exclusion from hypoxic tumor regions.

HIF-1α promotes the recruitment and polarization of immunosuppressive cell populations, including Tregs and MDSCs. Tregs, characterized by the expression of FoxP3, suppress effector T cell activity through the production of inhibitory cytokines such as IL-10 and transforming growth factor-beta (TGF-β) (67, 68). Hypoxia enhances Treg infiltration and stability via HIF-1α-induced CCL28 expression, which recruits Tregs to the tumor site. Similarly, MDSCs are recruited to the TME through HIF-1α-regulated chemokines, such as CXCL12. Once within the tumor, MDSCs exert potent immunosuppressive effects by producing arginase-1, ROS, and nitric oxide, which inhibit T cell activation and proliferation.

### 4.3 Role in resistance to immunotherapy

HIF-1α-mediated changes in the TME contribute significantly to resistance against immunotherapy, particularly ICIs. Clinical and preclinical studies have demonstrated that tumors with high HIF-1α activity exhibit poor responses to checkpoint inhibitors. For instance, patients with non-small cell lung cancer (NSCLC) and melanoma with hypoxic tumors are less likely to benefit from therapies involving anti-PD-1 or anti-PD-L1. This resistance is attributed to HIF-1α-driven upregulation of PD-L1, Treg accumulation, and CD8+ T cell exclusion from the TME. HIF-1α-induced metabolic changes, such as lactate production, further impair T cell function and survival. HIF-1α orchestrates multiple adaptations in the TME that render tumors refractory to immunotherapy. These include the remodeling of the ECM to create physical barriers against immune cell infiltration, the recruitment of immunosuppressive cell populations, and angiogenic induction that perpetuates vascular abnormalities and hypoxia. Collectively, these adaptations create an environment inherently resistant to T cell-mediated cytotoxicity and immune checkpoint blockade.

### 4.4 Therapeutic strategies targeting hypoxia inducible factor-1α

Given its central role in shaping the TME and mediating immunotherapy resistance, HIF-1α is an attractive therapeutic target. Several strategies have been demonstrated to inhibit HIF-1α activity or mitigate its effects on the TME. Combining HIF-1α inhibitors with ICIs has shown promise in preclinical models (69, 70). For instance, pharmacological inhibitors of HIF-1α, such as acriflavine and echinomycin, reduce PD-L1 expression, enhance CD8+ T cell infiltration, and improve the efficacy of anti-PD-1 therapies. These inhibitors disrupt the transcriptional activity of HIF-1α, thereby reversing hypoxia-induced immunosuppression. Additionally, combining HIF-1α inhibitors with adoptive T cell therapies or cancer vaccines can enhance immune cell trafficking and activation within the TME. Preclinical studies have demonstrated that targeting HIF-1α enhances the efficacy of these therapies and prevents the recruitment of immunosuppressive cell populations like Tregs and MDSCs.

Prodrugs such as evofosfamide are activated under hypoxic conditions, selectively targeting hypoxic tumor cells and not affecting normoxic tissues (71, 72). By reducing tumor hypoxia, HAPs indirectly inhibit HIF-1α activity and enhance the efficacy of ICIs. Agents such as hemoglobin-based oxygen carriers or perfluorocarbon emulsions can improve oxygen delivery to tumors, reducing hypoxia and HIF-1α stabilization (73). These treatments have improved immune cell infiltration and function within the TME. Therapies targeting VEGF or Ang-2 can normalize tumor vasculature, improving oxygenation and immune cell access to the tumor core. This approach has shown synergistic effects when combined with ICIs in preclinical studies. Nanotechnology has been employed to develop hypoxia-responsive drug delivery systems that target HIF-1α activity. For instance, nanoparticles loaded with HIF-1α inhibitors or oxygen-releasing agents can deliver their payload selectively to hypoxic tumor regions, minimizing off-target effects. These technologies in combination with ICIs and other immunotherapies, are being explored to enhance treatment outcomes.

HIF-1α plays a central role in shaping the TME to favor immune evasion and resistance to immunotherapy. By upregulating immune checkpoint molecules, suppressing effector immune cells, and recruiting immunosuppressive populations, HIF-1α undermines the efficacy of ICIs and other immunotherapies.

## 5 Potential interventions: targeting HIF-1α in the IVM

Targeting HIF-1α represents a promising strategy to overcome the immunosuppressive and tumor-promoting effects of the hypoxic IVM. A range of innovative approaches have been developed to inhibit the activity of HIF-1α, modulate the hypoxic TME, and enhance the efficacy of immunotherapy. These strategies include pharmacological inhibitors, gene editing technologies, hypoxia modulation, and nanotechnology-based delivery systems.

### 5.1 Pharmacological Inhibitors

Small molecule inhibitors targeting HIF-1α are being actively investigated as therapeutic agents in preclinical and clinical studies. These inhibitors aim to block the activity, stability, or transcriptional function of HIF-1α, thereby suppressing its downstream effects on angiogenesis, immune evasion, and metabolic reprogramming. Several compounds directly inhibit HIF-1α activity by interfering with its ability to bind DNA or dimerize with HIF-1β. Acriflavine, for example, disrupts the HIF-1α/HIF-1β dimerization, preventing the transcriptional activation of hypoxia-responsive genes (74, 75). Preclinical studies have demonstrated that acriflavine reduces tumor growth and improves the efficacy of checkpoint inhibitors by enhancing immune cell infiltration into the TME. Histone deacetylase (HDAC) inhibitors, such as vorinostat, have been shown to downregulate HIF-1α expression by altering chromatin accessibility (76, 77). These agents not only inhibit HIF-1α but also affect multiple pathways involved in immune regulation, making them attractive candidates for combination therapy. Agents targeting VEGF, a major downstream effector of HIF-1α, have been clinically validated in cancer therapy. Bevacizumab, a monoclonal antibody against VEGF, has shown efficacy in normalizing tumor vasculature and reducing hypoxia, indirectly inhibiting HIF-1α activity (78, 79). HAPs, such as evofosfamide, are designed to release cytotoxic agents specifically under hypoxic conditions. By selectively targeting hypoxic tumor regions, HAPs reduce the viability of HIF-1α-expressing cells, alleviating the hypoxic burden on the IVM (80, 81). Despite their promise, the clinical application of HIF-1α inhibitors faces challenges related to off-target effects, pharmacokinetics, and resistance mechanisms. Ongoing research aims to develop more selective and potent compounds to overcome these limitations.

### 5.2 Gene editing approaches

Gene editing technologies offer precise tools to target HIF-1α at the genomic level, providing a long-term solution to suppress its activity. Two major approaches, CRISPR-Cas9 and RNA interference (RNAi), have shown potential in preclinical studies. The CRISPR-Cas9 system allows the precise editing of the HIF-1α gene, leading to its permanent knockout. This approach effectively abolishes HIF-1α expression, preventing its stabilization under hypoxic conditions. In preclinical cancer models, CRISPR-mediated HIF-1α deletion has been shown to reduce tumor growth, inhibit angiogenesis, and enhance T cell-mediated immunity (82, 83). However, challenges related to delivery systems and off-target effects need to be addressed before clinical translation. RNAi-based approaches, including small interfering RNA (siRNA) and short hairpin RNA (shRNA), target HIF-1α mRNA to prevent its translation. siRNA nanoparticles targeting HIF-1α have demonstrated efficacy in reducing hypoxia-driven angiogenesis and immunosuppression in preclinical models. Advances in delivery technologies, such as lipid nanoparticles and conjugation with targeting ligands, have improved the stability and specificity of RNAi therapeutics. Gene editing technologies hold great promise for selectively targeting HIF-1α. However, issues related to delivery, immune activation, and potential off-target effects require further investigation to ensure safety and efficacy.

### 5.3 Modulating the hypoxic microenvironment

Modulating the hypoxic microenvironment is an indirect but effective approach to inhibit HIF-1α activity. By alleviating hypoxia, these strategies reduce the stabilization of HIF-1α and its downstream effects on the IVM. Oxygen carriers, such as perfluorocarbon emulsions and hemoglobin-based oxygen carriers, enhance oxygen delivery to tumors, mitigating hypoxia and reducing HIF-1α activity (73, 84). Preclinical studies have shown that these carriers improve immune cell infiltration and function, enhancing the efficacy of checkpoint inhibitors and adoptive cell therapies. Vascular normalization aims to restructure abnormal tumor vasculature to improve perfusion and oxygenation. Anti-angiogenic agents targeting VEGF, such as bevacizumab, have demonstrated the ability to transiently normalize blood vessels, reducing hypoxia and improving immune cell access to the tumor. Ang-2 inhibitors have also shown promise in promoting vascular stabilization and enhancing the delivery of immunotherapeutic agents. HBOT involves exposing patients to high-pressure oxygen, increasing oxygen levels in the bloodstream and improving tissue oxygenation (85, 86). By reducing tumor hypoxia, HBOT has been shown to enhance the efficacy of immunotherapy in preclinical models. Modulating the hypoxic microenvironment provides a complementary approach to direct HIF-1α inhibition, addressing the root cause of hypoxia-driven immunosuppression and therapeutic resistance.

### 5.4 Nanotechnology and drug delivery systems

Nanotechnology offers innovative solutions for delivering therapeutics to hypoxic tumor regions with high specificity and minimal off-target effects. Hypoxia-responsive nanoparticles have been developed to deliver HIF-1α inhibitors, siRNA, or oxygen-releasing agents selectively to the TME. These nanoparticles are designed to release their payload in response to hypoxic conditions. For example, nanoparticles loaded with acriflavine or siRNA targeting HIF-1α are coated with hypoxia-sensitive polymers that degrade under low oxygen levels, ensuring targeted delivery to hypoxic tumor cells (87, 88). Nanoparticles loaded with oxygen-generating agents, such as calcium peroxide or catalase, release oxygen into the TME, alleviating hypoxia and reducing HIF-1α stabilization. These particles have been shown to enhance immune cell infiltration and improve the efficacy of checkpoint inhibitors in preclinical models. Multifunctional nanoparticles capable of delivering multiple agents simultaneously have been developed to target multiple aspects of the hypoxic TME. For instance, nanoparticles combining HIF-1α inhibitors, ICIs, and oxygen-releasing agents have demonstrated synergistic effects in reducing tumor growth and enhancing immune responses. Nanotechnology-based delivery systems offer a versatile platform for targeting HIF-1α and modulating the hypoxic TME. Ongoing research focuses on optimizing their design, biocompatibility, and scalability for clinical use.

Targeting HIF-1α in the IVM offers a multifaceted approach to overcoming hypoxia-driven immunosuppression and therapeutic resistance in cancer. Pharmacological inhibitors, gene editing technologies, hypoxia-modulating treatments, and nanotechnology-based delivery systems represent complementary strategies to inhibit HIF-1α activity and enhance immunotherapy outcomes. While significant progress has been made, challenges related to delivery, specificity, and safety remain to be addressed. Continued research and innovation in these areas hold the potential to transform the landscape of cancer treatment by leveraging the full therapeutic potential of HIF-1α targeting interventions.

## 6 Challenges and future directions

The role of HIF-1α in the IVM presents both challenges and opportunities for advancing cancer immunotherapy. Successfully targeting HIF-1α requires addressing the complexity of the IVM, developing reliable biomarkers, integrating multi-omics approaches, and tailoring treatments to individual patients. This section explores these challenges and outlines potential future directions.

### 6.1 Complexity of IVM dynamics

The IVM is a dynamic and multifaceted system composed of immune cells, vascular structures, and stromal components that are interconnected through intricate signaling networks. This interdependence creates a highly heterogeneous microenvironment that is further shaped by HIF-1α activity and hypoxia. The immune, vascular, and stromal elements of the IVM are deeply intertwined. Hypoxia-induced HIF-1α activation promotes vascular abnormalities, including disorganized and leaky blood vessels, which impede immune cell infiltration and nutrient delivery. These changes further amplify hypoxia and perpetuate HIF-1α activity. Similarly, stromal cells such as cancer-associated fibroblasts (CAFs) produce ECM components that act as physical barriers to immune cells while sequestering growth factors like VEGF, exacerbating the immunosuppressive environment (89, 90). Spatial and temporal heterogeneity within the IVM adds another layer of complexity. Hypoxic regions often exclude cytotoxic T cells while fostering the accumulation of Tregs and MDSCs. This uneven distribution of immune cells poses significant challenges for the efficacy of immunotherapies, which rely on the ability to penetrate the entire TME. Addressing these challenges will require advanced models that accurately reflect the complexity of the IVM, such as 3D tumor organoids, organ-on-a-chip systems, and *in vivo* imaging technologies.

### 6.2 Biomarker development

Biomarkers are essential for predicting responses to therapies targeting HIF-1α and hypoxia-driven processes in the IVM. The identification and validation of reliable biomarkers can enable patient stratification, monitoring of therapeutic efficacy, and the design of tailored combination therapies. Hypoxia-associated gene signatures represent one avenue for biomarker development. HIF-1α regulates a suite of genes involved in angiogenesis, metabolism, and immune modulation, such as VEGF, GLUT1, and PD-L1. These gene signatures may provide insights into the activity of HIF-1α and hypoxia-related pathways, but tumor-specific panels are needed to account for variability across cancer types. Imaging-based biomarkers provide a non-invasive means to assess hypoxia and vascular abnormalities. Techniques like positron emission tomography (PET) and magnetic resonance imaging (MRI) using hypoxia-specific radiotracers, such as [18F] FMISO, have demonstrated utility in visualizing hypoxic regions within tumors. These imaging biomarkers could help guide patient selection for hypoxia-targeted therapies. Circulating biomarkers detected in liquid biopsies offer a minimally invasive alternative for monitoring HIF-1α activity. Circulating levels of VEGF, lactate, and soluble PD-L1, as well as exosomes containing HIF-1α-regulated transcripts, have shown promise in reflecting the state of the IVM. However, standardized assays and large-scale validation studies are needed to translate these biomarkers into clinical practice.

### 6.3 Integration with multi-omics approaches

The integration of genomics, proteomics, metabolomics, and transcriptomics provides a powerful framework for decoding the complexities of the IVM. Multi-omics approaches can uncover novel insights into the molecular mechanisms underlying HIF-1α activity and its impact on the TME. Genomics enables the identification of mutations and epigenetic changes that influence HIF-1α activity. For example, mutations in the VHL gene, which destabilize HIF-1α, are common in renal cell carcinoma and drive hypoxia-associated phenotypes. Genomic profiling can also reveal tumor-specific vulnerabilities that may be targeted therapeutically. Proteomics focuses on the downstream effectors of HIF-1α, including secreted factors like VEGF and interleukins that shape the IVM. Advanced mass spectrometry techniques have been used to identify post-translational modifications of HIF-1α, such as hydroxylation, which regulate its stability and activity. Metabolomics sheds light on the metabolic reprogramming induced by HIF-1α, such as the shift to glycolysis and lactate production. Elevated levels of lactate and other metabolites associated with hypoxia have been linked to immune suppression and therapeutic resistance, offering potential targets for intervention. The integration of multi-omics data through computational modeling and machine learning can identify critical pathways and networks driving HIF-1α activity in the IVM. These approaches hold the potential to uncover novel biomarkers and therapeutic targets that might not be evident from individual datasets.

### 6.4 Personalized medicine

Personalized medicine represents the future of cancer treatment, offering the potential to tailor immunotherapy strategies based on the unique characteristics of a patient’s IVM (91). This approach seeks to optimize therapeutic outcomes by accounting for the heterogeneity of the TME. Patient stratification is a cornerstone of personalized medicine. Biomarkers that reflect hypoxia, HIF-1α activity, and immune cell composition can be used to classify patients into subgroups most likely to benefit from specific therapies. For instance, patients with high HIF-1α and PD-L1 expression may respond well to a combination of HIF-1α inhibitors and ICIs. Combination therapies can be designed to address multiple aspects of the IVM simultaneously. Combining HIF-1α inhibitors with angiogenesis blockers, metabolic modulators, or adoptive T cell therapies can synergistically enhance anti-tumor responses. For example, targeting HIF-1α alongside VEGF inhibition may normalize tumor vasculature, improving immune cell infiltration and therapy delivery. Adaptive therapy strategies, which adjust treatment regimens in response to dynamic changes in the TME, are another promising avenue. By monitoring biomarkers during treatment, clinicians can modify therapeutic approaches to counteract resistance mechanisms and maintain efficacy. Artificial intelligence (AI) can play a critical role in personalized medicine by analyzing complex datasets and predicting treatment outcomes. AI-driven algorithms can integrate multi-omics data, imaging results, and clinical parameters to guide therapeutic decision-making and optimize treatment regimens.

The challenges associated with targeting HIF-1α in the IVM highlight the complexity of this critical therapeutic axis. The interdependence of immune, vascular, and stromal components, the need for robust biomarkers, and the integration of multi-omics approaches emphasize the necessity of multidisciplinary strategies. Personalized medicine, supported by advances in biomarker development and adaptive therapy approaches, represents a promising pathway for overcoming these challenges. By addressing these issues, researchers and clinicians can unlock the full potential of targeting HIF-1α to improve immunotherapy outcomes and patient survival.

## 7 Conclusion

HIF-1α occupies a central role in cancer biology, exerting profound effects on tumor progression and the IVM. Its stabilization under hypoxic conditions orchestrates a suite of adaptations that enable tumors to thrive in oxygen-deprived environments while evading immune surveillance. HIF-1α’s dual role in promoting tumor progression and reshaping the IVM underscores its significance as both a mechanistic driver of cancer pathology and a promising therapeutic target.

HIF-1α plays a pivotal role in facilitating tumor growth by adapting cellular and microenvironmental processes to low oxygen conditions. Through its regulation of genes involved in angiogenesis, metabolism, and immune modulation, HIF-1α drives critical aspects of tumor progression. For example, by upregulating VEGF, HIF-1α promotes the formation of abnormal, leaky blood vessels that sustain tumor growth but also exacerbate hypoxia. This self-perpetuating cycle of vascular dysfunction and hypoxia amplifies the tumor’s ability to resist immune attack and therapeutic intervention. Beyond its direct effects on tumor cells, HIF-1α profoundly influences the IVM by fostering an immunosuppressive environment. It upregulates immune checkpoint molecules such as PD-L1, leading to T cell exhaustion and impaired cytotoxic responses. Moreover, HIF-1α promotes the recruitment and polarization of immunosuppressive cells, including Tregs and MDSCs, while inhibiting the infiltration and activity of effector immune cells like cytotoxic T lymphocytes (CTLs) and NK cells. This immunosuppressive landscape, coupled with HIF-1α-driven ECM remodeling, creates physical and biochemical barriers that further protect tumors from immune-mediated destruction. The dual role of HIF-1α—as a driver of tumor progression and a mediator of immune suppression—positions it as a central player in the interplay between cancer cells and their microenvironment. Its broad range of effects highlights its potential as a critical target for therapeutic intervention.

Given its multifaceted role in tumor biology, HIF-1α represents an attractive target for therapeutic strategies aimed at enhancing immunotherapy outcomes. Current immunotherapies, such as ICIs, CAR-T cell therapy, and cancer vaccines, often face challenges due to the hypoxic and immunosuppressive conditions in the IVM. By targeting HIF-1α or its downstream pathways, these challenges can be mitigated, creating a more favorable environment for immune-based interventions. Combination therapies that integrate HIF-1α inhibitors with existing immunotherapies offer a promising strategy to enhance efficacy. For example, combining HIF-1α inhibitors with anti-PD-1/PD-L1 checkpoint inhibitors can reduce immunosuppression and improve T cell infiltration into tumors. Similarly, HIF-1α inhibition can synergize with CAR-T cell therapies by improving immune cell trafficking and survival within the hypoxic TME. Hypoxia modulation strategies, such as the use of oxygen carriers and vascular normalization agents, indirectly target HIF-1α activity by alleviating tumor hypoxia. These approaches not only reduce the stabilization of HIF-1α but also restore immune cell function, enhance drug delivery, and improve the overall efficacy of immunotherapies.

Advanced drug delivery systems, particularly those leveraging nanotechnology, offer precise and efficient methods for targeting HIF-1α. Hypoxia-responsive nanoparticles can selectively deliver therapeutic agents to hypoxic regions, minimizing off-target effects and maximizing therapeutic impact. Combining these delivery systems with immunotherapies can produce synergistic effects, improving both efficacy and safety. Despite its promise as a therapeutic target, challenges remain in effectively targeting HIF-1α. These include potential off-target effects, resistance mechanisms, and the difficulty of disrupting its transcriptional activity without affecting other critical pathways. Addressing these challenges will require innovative approaches and interdisciplinary collaboration.

Targeting HIF-1α and the hypoxic IVM requires a comprehensive understanding of the interplay between hypoxia biology and immune responses. Achieving this goal necessitates interdisciplinary research that bridges the fields of tumor biology, immunology, and bioengineering. A deeper understanding of the IVM’s complexity is essential for designing effective therapies. The IVM is a highly dynamic and heterogeneous environment that integrates immune cells, vasculature, and stromal components. Advanced 3D models, imaging technologies, and computational simulations can provide valuable insights into the spatial and temporal dynamics of these interactions under hypoxic conditions.

Biomarker development is another critical area for advancing therapies targeting HIF-1α. Reliable biomarkers for HIF-1α activity and hypoxia-driven immunosuppression are essential for patient stratification and monitoring therapeutic efficacy. Biomarkers derived from multi-omics approaches, imaging modalities, and liquid biopsies can guide the selection of patients most likely to benefit from HIF-1α-targeted therapies and enable real-time assessment of therapeutic responses. Integrating multi-omics data offers powerful tools for uncovering the molecular underpinnings of HIF-1α-driven changes in the IVM. Genomics, proteomics, metabolomics, and transcriptomics can reveal novel therapeutic targets, predict resistance mechanisms, and inform the design of personalized treatment strategies. Computational modeling and machine learning can further integrate these data streams to identify critical pathways and networks. Personalized and adaptive therapies are the future of immunotherapy. Tailoring treatments to the unique characteristics of each patient’s IVM can optimize therapeutic outcomes while minimizing adverse effects. Adaptive therapies that adjust regimens in response to real-time biomarker data will be particularly valuable for countering the dynamic and evolving nature of the IVM.

HIF-1α serves as both a driver of tumor progression and a mediator of immune suppression, making it a critical target for therapeutic intervention. Its role in regulating angiogenesis, immune evasion, and metabolic reprogramming highlights its central importance in shaping the IVM. Targeting HIF-1α through innovative approaches, such as combination therapies, hypoxia modulation, and advanced delivery systems, has the potential to enhance the efficacy of immunotherapies and overcome the challenges posed by the hypoxic TME. The complexity of the IVM demands interdisciplinary research that integrates insights from tumor biology, immunology, and bioengineering. By advancing our understanding of HIF-1α-driven processes and leveraging emerging technologies, researchers and clinicians can unlock new opportunities to improve cancer immunotherapy outcomes and patient survival. This integrated approach will pave the way for a new era of personalized and adaptive cancer treatment strategies that effectively target the hypoxic IVM.
